# Connection between hypoallergenic food and dyslipidemia in dogs: A nutritional myth?

**DOI:** 10.1371/journal.pone.0330556

**Published:** 2025-08-25

**Authors:** Andressa Rodrigues Amaral, Thiago Henrique Annibale Vendramini, Natália Manuela Cardoso de Oliveira, Laís Oyama Cotrim Lima, Pedro Henrique Marchi, Marcio Antonio Brunetto, Júlio Cesar de Carvalho Balieiro

**Affiliations:** 1 Veterinary Nutrology Service, Veterinary Teaching Hospital, School of Veterinary Medicine and Animal Science, University of Sao Paulo, Sao Paulo, Brazil; 2 Pet Nutrology Research Center, School of Veterinary Medicine and Animal Science, University of Sao Paulo, University of Sao Paulo, Pirassununga, Brazil; Faculty of Medical Sciences of Minas Gerais, BRAZIL

## Abstract

The limited education in nutrition contributes to the spread of the “nutritionism” phenomenon, with unfounded concerns about certain diets, such as commercial hypoallergenic ones, and their supposed relationship with the development of dyslipidemia. This study aimed to evaluate the perception of veterinarians on the subject and test its veracity. For this, serum triglyceride and cholesterol data from 35 dogs were analyzed before and after consumption of a commercial hypoallergenic diet for 60 days. In addition, a survey was carried out with veterinarians to assess their beliefs about this diet. Finally, the fat content of commercial maintenance, light and other prescription diets were compared to that of 10 commercial hypoallergenic dog diets. The results showed that most veterinarians believed that hypoallergenic diets could cause dyslipidemia and has too much fat. The analysis showed that hypoallergenic diets have fat content in the range of maintenance diets and majority of prescription diets. Fat content of hypoallergenic diets was only higher than light diets and supporting diets for diabetes available in Brazil. Laboratory analysis identified no dogs with dyslipidemia development during the study. Therefore, hypoallergenic diets do not have a high fat content, but new studies should verify if, when used for prolonged periods, hypoallergenic would lead to dyslipidemia in healthy dogs or dogs with underlying diseases, as well as possible changes in the lipoprotein profile.

## Introduction

Knowledge and teaching cat and dog nutrition has gained increased attention in recent years. However, surveys conducted with veterinarians show these professionals feel inadequately trained to deal with this subject, especially when it comes to interpreting food labels and prescribing nutritional management [1,2]. Additionally, many veterinarians do not trust information regarding commercial foods, as they consider this data may be biased by commercial interest [1]. Thus, the lack of knowledge from veterinary school, added to a lack of trust in the pet food industry may impair the dissemination of correct information among professionals. At the same time, many practitioners assume certain empiric results, stemming from unscientific anecdotal perceptions, as true.

Nutrition in general, in both human and animal health, is subject to various discussions and suffers with the dissemination of misinformation, as well as binary and reductionist recommendations that provoke fear and mistrust among the public. This phenomenon is often called “nutritional terrorism” or “nutritionism” by human health professionals and media outlets [3], and can cause severe impacts in patient well-being, such as depriving a patient of a potentially beneficial ingredient or diet due to false claims of deleterious effects. It is important for practitioners to watch for potential misinformation and to correct it as soon as possible.

The growing number of allergic patients in small animal medicine and the attempts to diagnose food hypersensitivity has led some professionals to speak against commercial hypoallergenic diets by claiming they may cause dyslipidemia, a common disorder in dogs and the majority of which are asymptomatic [4]. This study aims to evaluate the perception of veterinary practitioners about these diets and to check the veracity of this information. Moreover, we compared fat inclusion of commercial hypoallergenic and maintenance dog foods.

## Materials and methods

This study was carried out in agreement with the Ethical Principles in Animal Research established by the Ethic Committee on Animal Use of the School of Veterinary Medicine and Animal Science of the University of Sao Paulo (CEUA/FMVZ). The study was approved under protocol number 2084090323. Also, this study was approved to perform the survey with humans by the National Research Ethics Commission (CONEP) Brazil under the protocol 7.373.056.

Thirty-five dogs, castrated, male or female and of different breeds, from the routine veterinary teaching hospital of a university in the state of São Paulo, Brazil, were evaluated. Of these, sixteen dogs had pruritic dermatitis with or without skin lesions, and nineteen were healthy and were selected as the control group. All dogs ate the hypoallergenic supporting diet with hydrolyzed protein for 60 days (Table 1) with the amount calculated to achieve energy requirement for maintenance of inactive adult dogs 95 × body weight^0.75^ [5]. After stabilization of dermatological signs and treatment of associated infections, dogs in the AL group began a food trial lasting 8 weeks (60 days), followed by a food challenge with their previous diets (maximum 14 days).

**Table 1:** Nutritional content of the hypoallergenic diet with hydrolyzed protein.

CP	Moisture	Fat	Ash	TDF	ME
%	%	%	%	%	Kcal/kg
22	10	16	7.5	13.5	4060

CP: crude protein; TDF: total dietary fiber; ME: metabolizable energy

No animals had received commercial or homemade hypoallergenic diets, omega-3 supplementation, or oral corticosteroids or antibiotics for at least one month prior to and during the diagnosis period. All animals received appropriate prophylaxis against ectoparasites.

A blood sample was collected by venipuncture, after 12 hours of fasting, before (T0) and after (T60) the elimination period for analysis of the lipid profile (triglycerides and total cholesterol).

### Total cholesterol and triglycerides

Blood samples were collected to perform the lipid profile, by puncturing the jugular vein with a hypodermic needle and 3 ml or 5 ml syringes. After collecting, the blood was stored in a tube with clot activator and separating gel and then subjected to centrifugation at 3000 rotations per minute for 5 minutes. After separating the serum, it was transferred to Eppendorf tubes and stored at −20ºC until processing in the clinical analysis laboratory of the veterinary school hospital. For adult dogs, the reference values for cholesterol were 125–270 mg/dL and triglycerides 40–169 mg/dL, according to the values of the laboratory responsible for processing in this study.

### Research with veterinarians

With the Google Forms^®^ tool, an online survey with non-nutritionist veterinary practitioners was performed. The professionals were required to answer “yes” or “no” to the questions: “1- Can commercial hypoallergenic diets cause dyslipidemia?”; “2- Can commercial hypoallergenic diets cause pancreatitis?”; “3- Do commercial hypoallergenic diets have too much fat?”; and “4- Do commercial hypoallergenic diets cause problems in the long term?” To answer these questions, the veterinarians were asked to disregard one commercial hypoallergenic diet that had a “moderate calorie” claim. Nutritionists were not allowed to answer the questions.

### Comparison of fat content in commercial diets

Available data on inclusion of fat in dry and extruded commercial dog food was collected on the websites of eight manufacturers for super premium maintenance dog food and seven manufacturers for hypoallergenic dog food. For this analysis, the inclusion was converted to “g/1000kcal” based on the metabolizable energy declared by the manufacturer or, when unavailable, calculated by the modified Atwater equation [5]. One commercial hypoallergenic diet that already claimed to be moderate in fat/calorie (“moderate calorie”) was excluded from the analysis since it already has lower fat content on purpose.

Additionally, the fat content of Hypoallergenic diets was also compared to other dog’s supporting diets who does not have a claim of being low in fat inclusion and have other nutrient restrictions: diets for kidney disease (n = 9), cardiac disease (n = 4), urinary disease (n = 8), gastrointestinal except moderate calorie, low fat and puppy (n = 9), diabetes (n = 7). And, finally, supporting diets with a “moderate calorie” (n = 2) (1 hypoallergenic and 1 gastrointestinal) or “low fat” (3 gastrointestinal) claim were compared with light maintenance super premium dog foods (n = 18).

To access the representative value of metabolizable energy (ME) from fat, the value obtained in g/kg of fat was multiplied by 9.4 (Atwater equation) and then calculated as a percentage of the total ME of the label or calculated [5].

### Statistical analysis

At the end of the experimental period, the results of triglycerides and cholesterol were analyzed using their means, considering the effect of treatment, time and interaction *versus* treatment. Statistical analyzes were performed using the Student’s t-test for paired data (T before versus T after) and the T-test for unpaired data for variables that met data normality assumptions. Variables that did not meet this assumption were analyzed using the non-parametric Wilcoxon test. The Tukey test was applied and values of p < 0.05 were considered significant [6]. All analyses were performed using the Statistical Analysis System program (SAS Institute Inc., Cary, NC, USA).

For the remaining variables, a descriptive statistic considering the mean, standard deviation, minimum (min) and maximum (max) values as well as representative percentages were calculated and compared numerically.

## Results

The survey collected 53 responses from non-nutritionist veterinarians, who responded affirmatively to the four questions addressed, as shown in Table 2 below.

**Table 2:** Total affirmative answers to the questions on the online form linked to veterinarians from different specialties.

	Questions	Total
1	Can commercial hypoallergenic diets cause dyslipidemia?	54.7%
2	Can commercial hypoallergenic diets cause pancreatitis?	32.1%
3	Do commercial hypoallergenic diets have a lot of fat?	60.4%
4	Do commercial hypoallergenic diets cause long-term problems?	28.2%

Regarding data on fat inclusion in commercial dog foods, all data were analyzed including variations for different dog sizes and resulted in 78 dry super premium maintenance foods. Mean fat inclusion for these foods was 38.18 ± 4.51g/1000kcal (min. 27.78, max. 48.19g/1000kcal). For the hypoallergenic dry dog foods, 15 diets were analyzed, and mean fat inclusion was 34.98 ± 5.02g/1000kcal (min. 26.46, max. 41.76g/1000kcal).

**Fig 1:**
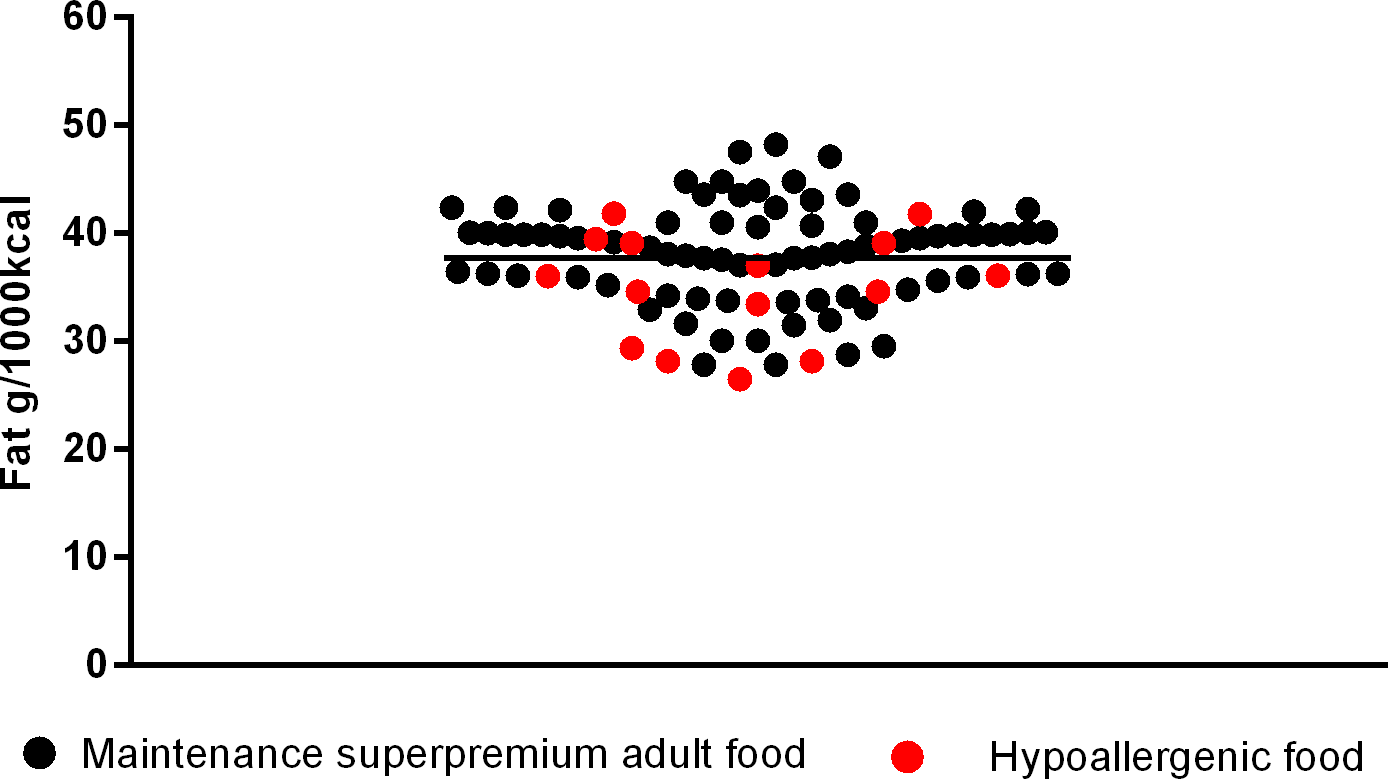
Fat content in super premium maintenance and hypoallergenic dog food.

Moreover, it was observed that 78.21% of the maintenance diets had fat inclusion between the minimum and maximum values found in hypoallergenic diets, and the remaining 21.79% of maintenance diets had fat inclusion above the maximum found in hypoallergenic diets (Fig 1). Additionally, the mean fat inclusion for hypoallergenic diets was 3.20%, lower than the mean for maintenance diets.

Among supporting renal diets, mean fat inclusion was 42.24 ± 3.01g/1000kcal (min. 36.13 and max. 46.32 g/1000 kcal); for cardiac support the mean fat was 43.42 ± 1.89g/1000kcal (min. 41.34 and max. 45.92g/1000kcal); for urinary supporting diets, the mean was 39.05 ± 6.96g/1000kcal (min. 25.41 and max. 47.15g/1000kcal); for gastrointestinal diets was 35.99 ± 6.02 (min. 27.05 and max. 43.75g/1000kcal) and for diabetes support was 28.55 ± 6.40g/1000 kcal (min. 20.15 and max. 43.55g/1000 kcal). Finally, the mean fat inclusion for maintenance super premium light foods for dogs was 30.18 ± 4.17g/1000 kcal (min. 22.79 and max. 36.43g/1000 kcal).

The hypoallergenic diet with a “moderate calorie” claim had a maximum fat content declared on the label of 35.57g/1000kcal, the low-fat gastrointestinal diets had a mean of 17.10 ± 3.59 g/1000kcal of fat (min. 14.56 and max. 21.21g/1000kcal). As for the representative fraction (%) of fat in total ME, the average and range was: maintenance 35.89 ± 3.37 (min. 26.11 and max. 45.30), light 25.61 ± 3.37 (min. 21.15 and max. 31.16), hypoallergenic 32.88 ± 4.72 (min. 24.87 and max. 39.25), renal 39.88 ± 2.83 (min. 33.96 and max. 43.54), cardiac 40.81 ± 1.54 (min. 38.86 and max. 43.16), urinary 36.70 ± 6.54 (min. 23.88 and max. 44.32), gastrointestinal 33.83 ± 5.66 (min. 25.43 and max. 41.13) and diabetes support 26.84 ± 7.76 (min. 18.94 and max. 40.94).

**Fig 2:**
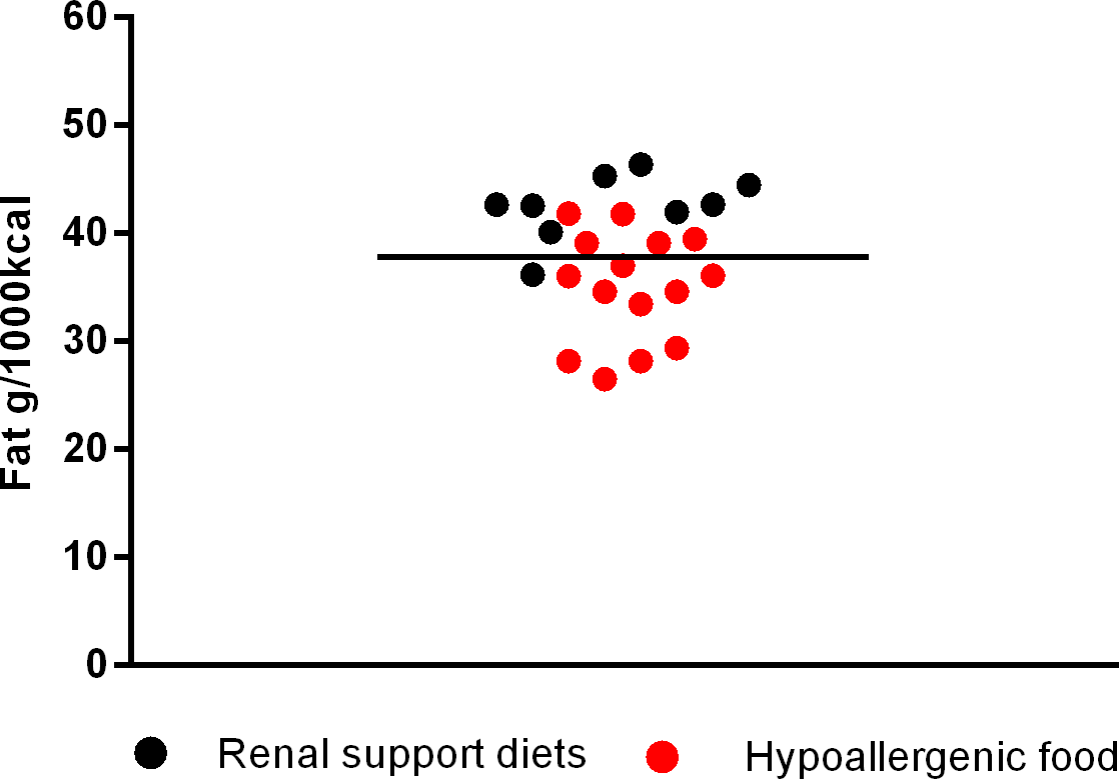
Fat content in renal support diets and hypoallergenic dog food.

**Fig 3:**
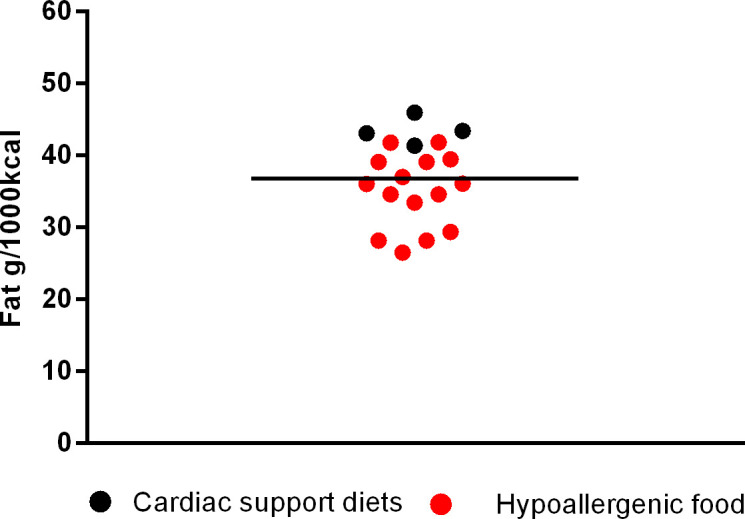
Fat content in cardiac support diets and hypoallergenic dog food.

**Fig 4:**
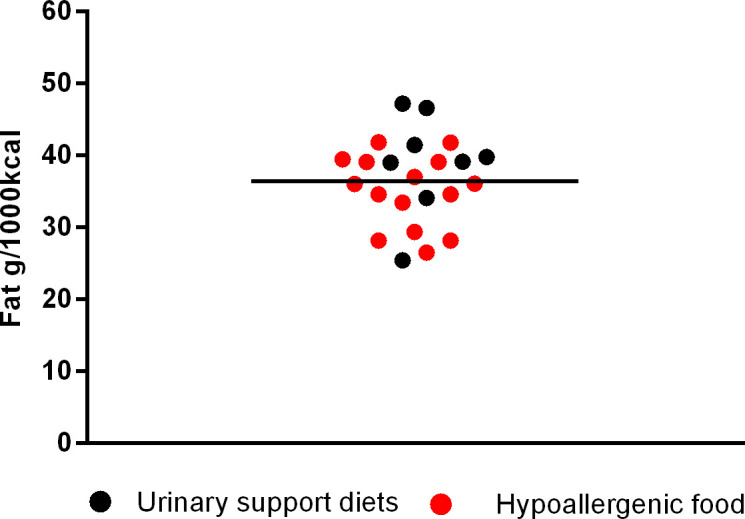
Fat content in urinary support diets and hypoallergenic dog food.

**Fig 5:**
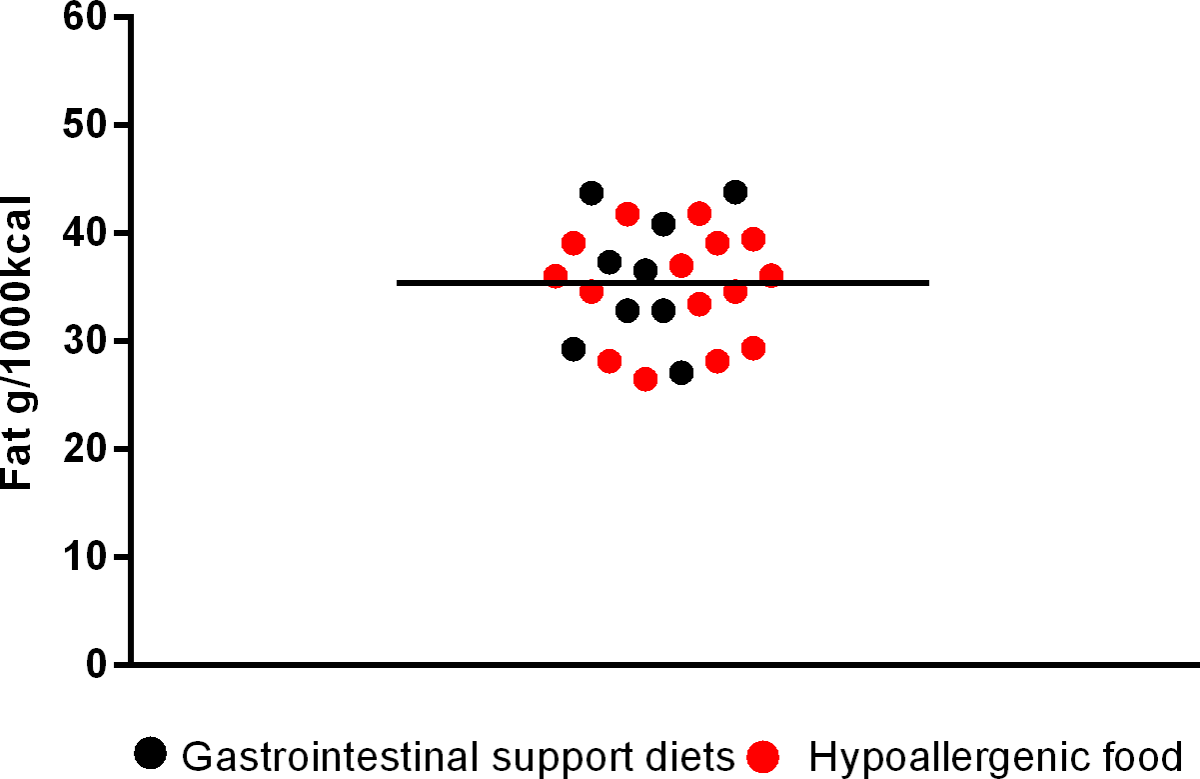
Fat content in gastrointestinal support diets and hypoallergenic dog food.

**Fig 6:**
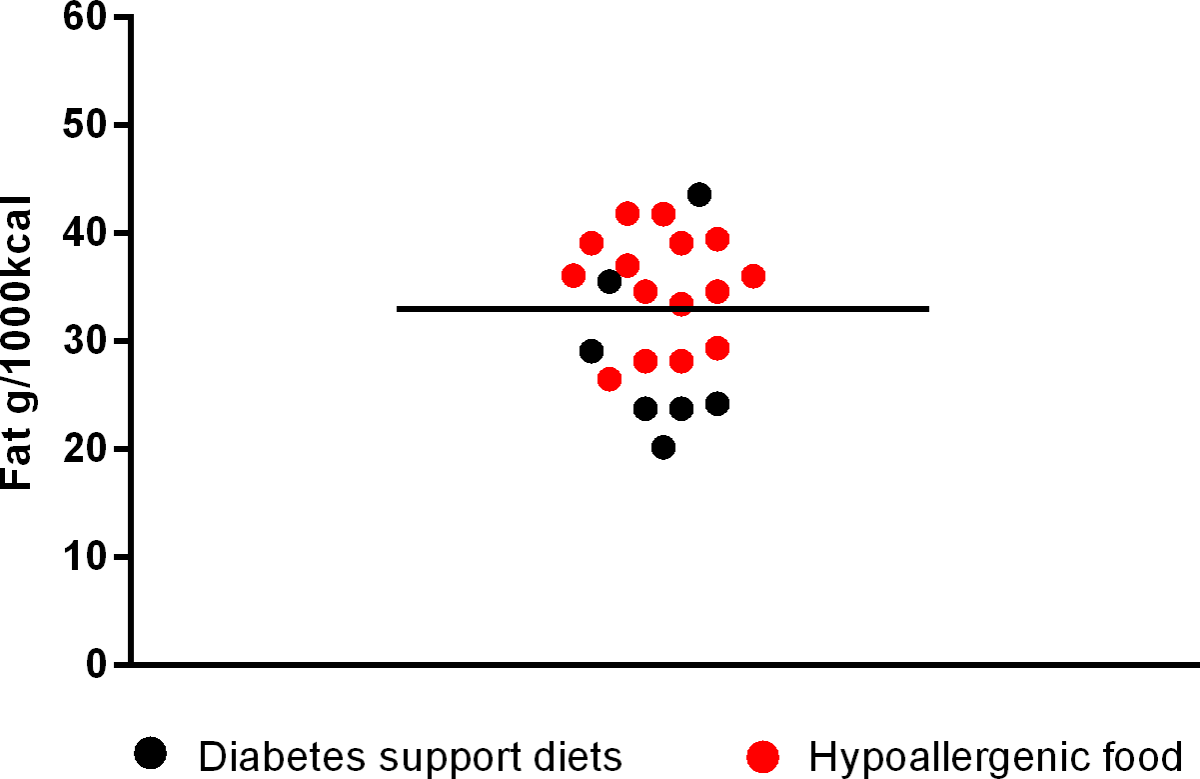
Fat content in diabetes support diets and hypoallergenic dog food.

**Fig 7:**
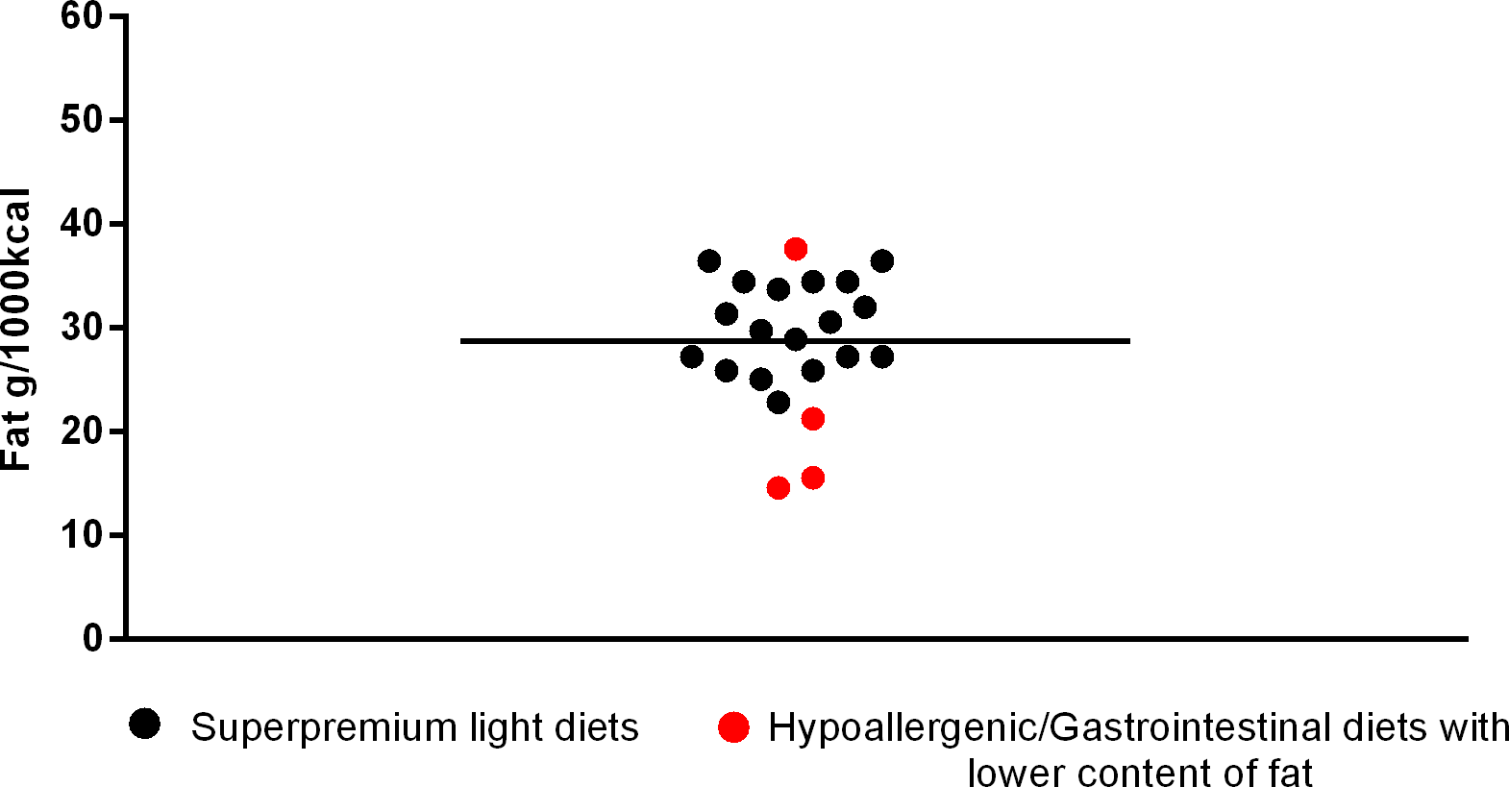
Fat content in light superpremium diets and hypoallergenic or gastrointestinal diets with lower fat.

**Fig 8:**
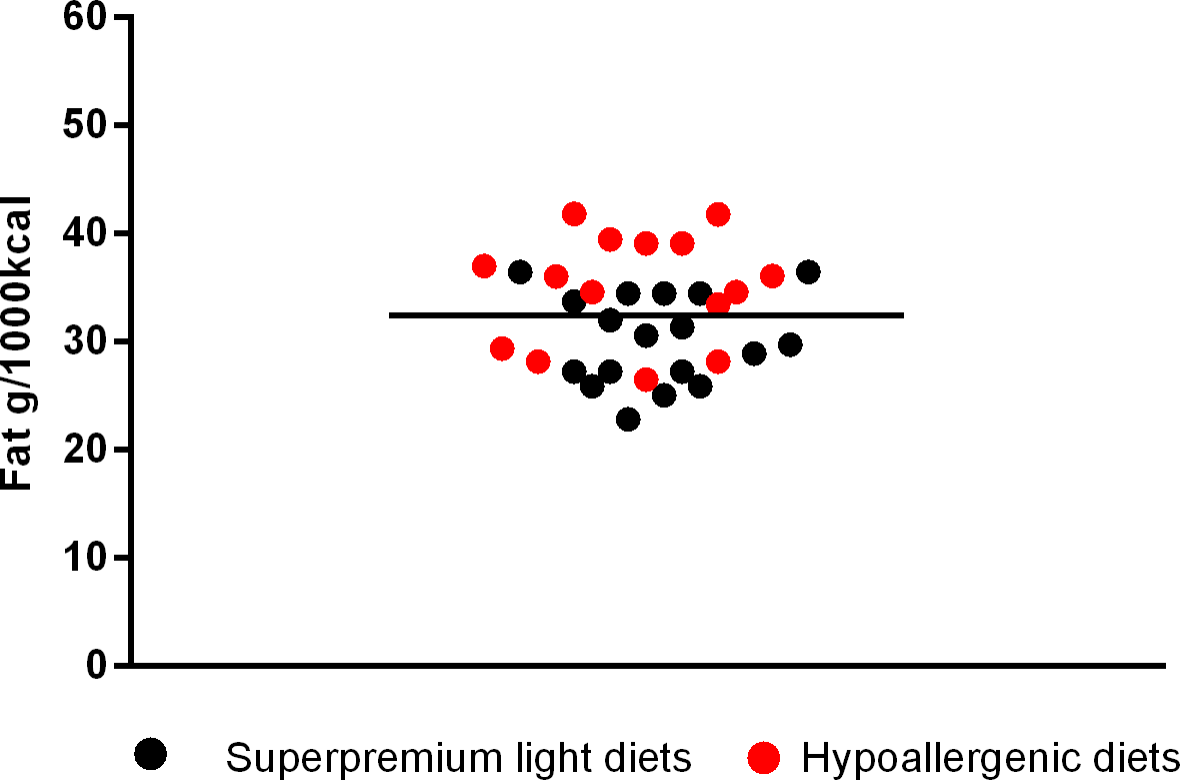
Fat content in light superpremium diets and hypoallergenic diets.

The next Figs 2–6 shows the distribution of the fat content of hypoallergenic diets among other supporting diets and Fig 7 shows the distribution of the lower fat content diets (diets with this specific claim) among the light diets and Fig 8 shows the fat content distribution between hypoallergenic diets and light super premium diets.

By comparing hypoallergenic diets with other supporting diets, the average fat was lower than the averages found in renal, cardiac, urinary and gastrointestinal diets. Also 3 out of 15 had lower fat than the average fat of diabetes diets, being one from the same brand and type but formulated for different dog sizes. Moreover 9/15 had a lower fat content than the maximum value found in a light diet (<36.43g/1000kcal) – with two of them being only a variation of the dog size.

After a period of 60 days of using a hypoallergenic diet, the evaluation of serum triglycerides and cholesterol (Table 3) in animals without previous changes in these settings showed no significant changes, remaining within the reference range for the species. It was necessary to increase the energy requirement for some dogs that lost weight, in those cases, energy requirement for active adult dogs was used [5].

**Table 3:** Mean serum concentration of triglycerides and cholesterol (mg/dL) and standard deviation before (T0) and after (T60) food trial.

Variable	Time		P-value		
	T0	T60	Tt.	Time	Tt. x Time
Triglycerides	80.40 ± 59.12	66.97 ± 39.75	0.62	0.24	0.79
Cholesterol	211.89 ± 51.60	239.89 ± 59.82	0.79	0.04	0.55
Tt. = treatment.					

All diets, apart from light formulations, had a maximum fat content higher than the maximum found in hypoallergenic foods. Therefore, hypoallergenic diets do not represent the group with the greatest fat inclusion. It is important to state that, at the time of this research, it was found inconsistency between the fat content reported on the website and the official catalog of products of a pet food company. We used the information from the catalogue as a basis since it was declared on the original basis and the website in dry matter, and the fat content in the catalog was lower. After 60 days of the tested hypoallergenic diet intake, triglycerides reduced, however with no statistical significance and cholesterol increased about 28 mg/dL, although remaining within reference values.

## Discussion

The food trial is considered the gold standard for diagnosis of food allergy, which may be accountable for up to 10% of allergic animals [7]. The therapeutic strategy consists in providing unconventional or hydrolyzed proteins, aiming to avoid mast cell degranulation and the release of proteases and histamine [7].

The pathogenesis of food allergy is still not completely understood. However, the current literature recommends a formulation with the minimum supply of protein, as protein is the responsible for the allergic reaction, even when hydrolyzed or when it is unconventional according to the patient’s dietary history [7]. Thus, other macronutrients, such as fat, may occupy the subtracted percentage of protein.

Analysis shows hypoallergenic fat content is still among usual values found in commercial maintenance diets, meaning that the 60% of practitioners that agreed with the sentence “Do commercial hypoallergenic diets have a lot of fat?” are wrong. It is even possible to say that, depending on the products, the switch from a maintenance diet with the highest fat declared (48.19g/1000kcal) to a hypoallergenic diet with the lowest fat declared (26.46g/1000 kcal) may represent a reduction in fat ingestion by almost 55%.

The difference between energy content and moisture of diets may be one of the factors that hinder veterinarians’ understanding of pet food labels. Consequently, relying solely on fat percentages is inaccurate, as comparison should be made using a consistent basis, either dry matter basis or in g/1000 kcal or to calculate the exact fat daily fat intake considering all that is consumed in a day, which is something that is not commonly done in routine clinical practice.

The only type of diets where hypoallergenic diets had overall higher fat was in the light group, which is expected to, since their formula aims specifically in controlling fat and energy. Even though, 9 out of 15 hypoallergenic diets had lower fat than the maximum fat content found in light diets (less than 36.43g/1000kcal). This is possible because the term “light” refers to products that have undergone a reduction of at least 15% in energy density compared to a complete maintenance food for adult animals within the same brand [8]. There is no global recommendation for the appropriate range of fat that a light diet should have.

In this study, an important limitation is that the fat content of most diets is declared as being the minimum. To minimize this problem, when minimum and maximum were declared, the maximum value was used for the analysis. However, no hypoallergenic diet declared its maximum values of fat. The only one to do so was that with the claim of being lower in fat since it is a regulatory labeling demand. For these, their fat content resembles the fat content of “light” foods.

A more appropriate approach would have been to perform a bromatological analysis [5,9] which was not done in this work. However, considering that fat is typically one of the most expensive ingredients in super premium formulations [10], it is unlikely that the actual fat content is significantly higher than the value declared.

Regarding those practitioners who agreed with the sentence: “Can commercial hypoallergenic diets cause dyslipidemia”, the evaluation of serum triglycerides and cholesterol emphasize that animals without previous alterations in these parameters remained within values of reference for the species after the 60-day period. There was an increase in cholesterol levels, although within the range of normality and biologically not significant despite the numeric result. Further studies should investigate this in the long term, first in healthy subjects. Therefore, until this moment, these practitioners do not have any reason to believe that these diets cause dyslipidemia. The number of subjects submitted to analysis is also a limitation of our study, and future research should aim to investigate larger populations.

Healthy dogs are known as HDL-positive species (4). This means that a rise in cholesterol is not necessarily detrimental for them. Therefore, in addition to long-term investigation, it is also important to evaluate the lipoprotein profile. Another concern was the higher risk for developing pancreatitis, since 28.2% agreed with the sentence “Can commercial hypoallergenic diets cause pancreatitis”. No clinical signs of pancreatitis were seen in our patients.

Cridge et al. [11] reviewed the etiology and risk factors of pancreatitis in dogs and stated that fat itself is not a single risk factor for pancreatitis. When it comes to diet effect and fat content consumption, it will most likely rely on the presence of hypertriglyceridemia (that means that the patient has alterations in fat metabolism) but not cholesterol, and obesity, increasing by 1,3 times the risk for acute pancreatitis in this case. The authors also criticized the diagnosis of pancreatitis in previous studies that suggested a possible interpretation that fat content may have caused it. Also, James et al., [12] showed in dogs that pancreatic lipase immunoreactivity results were not changed in healthy dogs eating a diet with 16% or 5% fat.

Xenoulis et al., [13] found that more than 70% of the dogs with naturally occurring pancreatitis had cholesterol and triglycerides within the range of normality, and when high triglycerides were found, it was mild. They observed that these patients had higher concentrations of low-density lipoprotein (LDL) and lower high-density lipoprotein (HDL) than healthy subjects even when triglycerides and cholesterol were normal. Therefore, the intrinsic metabolic differences that lead to the formation of LDL and type of fatty acid intake could play a bigger role than the amount of fat itself. Therefore, the concern that veterinarians claim in developing pancreatitis following hypoallergenic diet consumption is not only unjustified by the fat content of hypoallergenic diets not being much different from the remaining diets in the market, but also by the etiology of the disease itself.

The effects in animals with previous dyslipidemia or predisposing diseases may be inquired and evaluated in the future. In fact, literature points that multimodal treatment, which allies reduction of fat ingestion and medication treatment, represents the best therapeutical protocol for the management of dyslipidemia [14,15]. The outcome for patients with underlying metabolic diseases such as hypercortisolism, hypothyroidism, or primary dyslipidemia may be different. However, it means that these patients require a specific diet in context of their disease, but not a problem with the hypoallergenic diets.

In this context, recommendations for diet modification are plausible for patients that consume maintenance diets or any other diets that do not have reduced fat. Hypoallergenic diet is not the sole diet to difficult dyslipidemia treatment. Furthermore, there are no references to determining what would be considered “high” or “low” dietary fat [11]. According to the results of this work, the only diets with lower fat content than hypoallergenic formulations were light, which are expected to, and diabetes support diets considering the average fat content. Therefore, in general, using a hypoallergenic diet for overweight animals or those with medical conditions that predispose to dyslipidemia (primary or secondary) would not be appropriate for the control of these conditions. However, it was never expected for these diets to do so and the new hypoallergenic diets with the lower fat content claim could also be a form of “light” version of a supporting diet, which in many cases can still be insufficient to control dyslipidemia without medication support.

It is also important to note that fat is the main source of energy for dogs [5] and could possibly indicate high quality according to the type of fatty acids content. Therefore, it is also not inadequate to include it in the referred amounts in super premium diets for dogs. Still, for overweight patients, the nutrient proportion may be directly related to this condition, as well as positive energy balance and, possibly, gut microbiota [16]. Nonetheless, no dogs in this study gained weight.

Nutritional orientation should mainly consider previous fat ingestion and promote a reduction based on this value. It is important to point out the need for veterinary practitioners to accustom themselves to correctly reading commercial food labels in every situation in which dietary change might be advised or send the patient to a nutritionist rather than simply rely on misinformation.

Even if the hypoallergenic diets could predispose to dyslipidemia, that would not be justified by a “high” fat content, as despite being similar to that of maintenance dog diets, they have lower fat content than other supporting diets that does not hold the same fame. Furthermore, diets considered “high fat” in experimental studies are those with 30% to 50% of metabolizable energy (ME) coming from fat in their composition [17,18], a characteristic found in hypoallergenic diets analyzed in this study, but also found in maintenance diets, renal, cardiac, urinary, gastrointestinal and diabetes. A possible way to overcome this problem of considering as “high fat” most of the diets that are not “light” or “restricted” in fat, would be finding an average fat content and consider it as “regular” amount of fat, since naming it as high or low is inaccurate.

Hsu et al. [19] observed differences in fat metabolites from dogs that consumed diets based on hydrolyzed chicken protein. These animals presented lower plasma concentrations of bile acids, and higher fecal concentrations of conjugated bile acids in comparison to non-conjugated. Contrary to what was believed, lipid digestion seemed to be facilitated, and lipid metabolism benefited by the ingestion of hydrolyzed protein, even though more studies would be needed.

Hydrolyzed protein also results in less undigested substrates, and thus, lower availability of those for undesired microbial fermentation and production of oxidative metabolites such as phenols. Pinto et al. [20] observed higher concentrations of fecal lactate in dogs receiving hydrolyzed protein, which is considered beneficial as it is produced by symbiotic bacteria [21].

Additionally, studies with animal models indicate that provision of hydrolyzed protein in the long term could contribute to anti-hypertensive effects [22,23]. This effect still lacks confirmation for dogs and cats, however, if proven true, may represent a point in favor for the long-term use of hypoallergenic diets based on hydrolyzed protein, especially for patients with endocrinopathies that predispose to hypertension, such as hypercortisolism. Further studies in this area are needed to clarify potential positive or negative effects in these species, as the specific concern raised in the survey regarding long-term outcomes still lacks sufficient scientific evidence.

Future studies could also investigate the role of fiber content, as these diets typically include fiber in standard maintenance amounts. Potential mechanisms of interest include the gel-forming capacity of soluble fibers which may promote bile acid retention. This, in turn, could increase the hepatic conversion of cholesterol for bile acid formation due to reduced bile acid recycling. Additionally, soluble fibers may reduce lipid absorption, retain glucose and decrease the lipogenesis *de novo* process, or even exert their effects through the short-chain fatty acids formation and their immunomodulatory effects, and lipid lowering properties by unknown mechanisms). Taken together, this may be an option for hypoallergenic diets formulations for dogs with underlying alterations in fat metabolism [24–27]

## Conclusions

Most veterinary practitioners of the analyzed sample believe that commercial hypoallergenic diets have high in fat and predispose to dyslipidemia. However, as demonstrated in this study, fat content in these diets is similar to maintenance diets. The ingestion of the hypoallergenic hydrolyzed diets for 60 days did not result in dyslipidemia in healthy and allergic dogs. More studies are necessary to evaluate this variable in the long term and in healthy patients and patients with underlying diseases, as well as the lipoprotein profile in both cases. Further studies should also evaluate the fat content through bromatological analysis for better accuracy since using the values declared on label is a limitation of our study. Hypoallergenic diets were safe from many perspectives. The concerns regarding these diets lack foundation and dietary changes require individual evaluations. Incorporation of veterinary nutritionists is indispensable in these situations when the clinician does not feel capable of making the required analyses.

## Supporting information

S1 File All diets (blind). (XLSX)
